# Typical ECG findings in an unconscious patient

**DOI:** 10.1007/s12471-016-0910-y

**Published:** 2016-10-26

**Authors:** R. Joustra, F. N. Polderman, J. L. Smeets, M. C. Daniëls, M. Boulaksil

**Affiliations:** 1 Department of Cardiology, Jeroen Bosch Hospital, ’s-Hertogenbosch, The Netherlands; 2 Department of Cardiology, Radboud University Medical Center, Nijmegen, The Netherlands; 3 Department of Intensive Care Medicine, Jeroen Bosch Hospital, ’s-Hertogenbosch, The Netherlands

## Answer

**Fig. 1:**
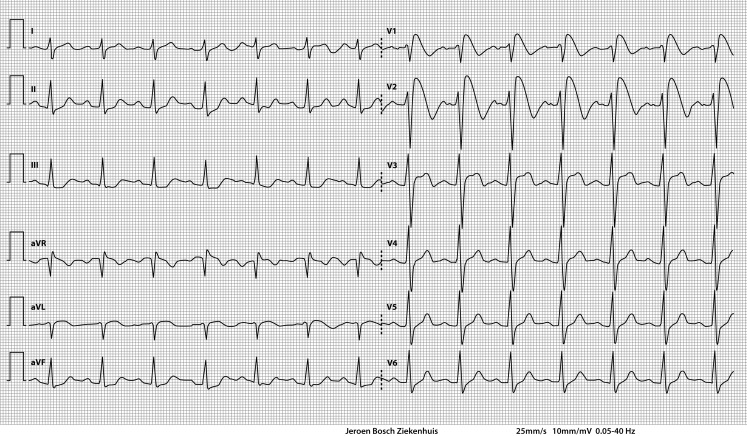
ECG on admission

Notably, the ECG on admission shows a Brugada-like electrocardiographic pattern (with right bundle branch block and typical ST-segment elevations in leads V1–V3 with a terminal negative T wave). This Brugada-like pattern is called coved type or type 1 (Fig. 1).

In individuals with a normal baseline ECG, this pattern can be provoked by controlled infusion of sodium channel blocking agents for the diagnosis of Brugada syndrome [1]. However, intoxication with a sodium channel blocking agent may give rise to a transient Brugada-like pattern without an underlying Brugada syndrome.

In patients with a psychiatric history, one should consider involvement of a tricyclic antidepressant (TCA), since TCAs are known to result in sodium channel blockade [2]. Our patient turned out to be using nortriptyline, a TCA, amongst other non-tricyclic antidepressants. Therefore, the suspicion of an intoxication with this drug was raised [3]. Indeed, blood tests showed toxic levels of nortriptyline (507 µg/l (therapeutic levels: 50–150 µg/l; toxic levels: >500 µg/l), E‑10-OH-nortriptyline 386 µg/l).

**Fig. 2:**
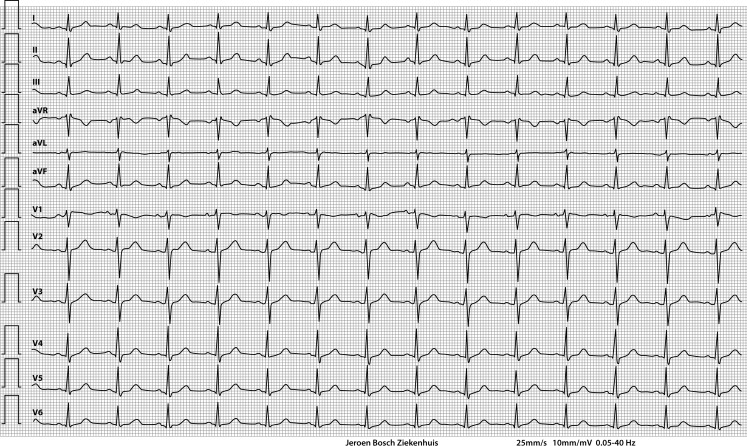
Follow-up ECG after discharge from the intensive care unit

She was admitted to the intensive care unit and treatment with activated charcoal was started to reduce enteral absorption. Furthermore, she was alkalinised with sodium bicarbonate infusion in order to increase binding of nortriptyline to serum proteins. On follow-up, the ECG abnormalities eventually dissolved (Fig. 2).

## Conclusion

Type 1 Brugada-like ECG pattern provoked by TCA intoxication.
